# Maternal microbiota-derived metabolic profile in fetal murine intestine, brain and placenta

**DOI:** 10.1186/s12866-022-02457-6

**Published:** 2022-02-07

**Authors:** Tiina Pessa-Morikawa, Aleksi Husso, Olli Kärkkäinen, Ville Koistinen, Kati Hanhineva, Antti Iivanainen, Mikael Niku

**Affiliations:** 1 Department of Veterinary Biosciences, Faculty of Veterinary Medicine, University of Helsinki, Helsinki, Finland; 2 School of Pharmacy, University of Eastern Finland, Kuopio, Finland; 3 Afekta Technologies Ltd., Kuopio, Finland; 4 Institute of Public Health and Clinical Nutrition, University of Eastern Finland, Kuopio, Finland; 5 Food Chemistry and Food Development Unit, University of Turku, Turku, Finland

**Keywords:** Metabolomics, Microbiota, Fetus, Placenta, Brain, Intestine, Prenatal, Development

## Abstract

**Background:**

The maternal microbiota affects the development of the offspring by microbial metabolites translocating to the fetus. To reveal the spectrum of these molecular mediators of the earliest host-microbe interactions, we compared placenta, fetal intestine and brain from germ-free (GF) and specific pathogen free (SPF) mouse dams by non-targeted metabolic profiling.

**Results:**

One hundred one annotated metabolites and altogether 3680 molecular features were present in significantly different amounts in the placenta and/or fetal organs of GF and SPF mice. More than half of these were more abundant in the SPF organs, suggesting their microbial origin or a metabolic response of the host to the presence of microbes. The clearest separation was observed in the placenta, but most of the molecular features showed significantly different levels also in the fetal intestine and/or brain. Metabolites that were detected in lower amounts in the GF fetal organs included 5-aminovaleric acid betaine, trimethylamine N-oxide, catechol-O-sulphate, hippuric and pipecolic acid. Derivatives of the amino acid tryptophan, such as kynurenine, 3-indolepropionic acid and hydroxyindoleacetic acid, were also less abundant in the absence of microbiota. Ninety-nine molecular features were detected only in the SPF mice. We also observed several molecular features which were more abundant in the GF mice, possibly representing precursors of microbial metabolites or indicators of a metabolic response to the absence of microbiota.

**Conclusions:**

The maternal microbiota has a profound impact on the fetal metabolome. Our observations suggest the existence of a multitude of yet unidentified microbially modified metabolites which pass through the placenta into the fetus and potentially influence fetal development.

**Supplementary Information:**

The online version contains supplementary material available at 10.1186/s12866-022-02457-6.

## Background

The intestinal microbiota has a great impact on the life and wellbeing of the host. Microbes residing in the gut participate in digestion and metabolic modification of nutrients, producing substances that are absorbed by the host [1]. The cell numbers of gut microbiota are estimated to at least equal and its gene pool exceed that of its host [2, 3]. The potential of the microbiota to influence host metabolism is illustrated by a comparison of the serum metabolome of conventionally colonized and germ-free (GF) mice: 3.5% of the > 4000 molecular features detected were unique for conventional mice and 10% of the shared molecular features had significantly different levels between the groups [4]. All the organ systems of the host are affected to a varying degree [5].

While a majority of the compounds originating from microbial metabolism detected in mammalian tissues still remain uncharacterized, some of these substances and their effects on the host are well documented. These include short chain fatty acids (SCFAs) which the host utilizes as an essential part of its metabolism [6]. The SCFAs produced from complex carbohydrates by microbes residing in the alimentary tract are an important source of energy for the host [7]. The SCFAs have also been shown to contribute to the maintenance of the gut epithelium and regulation of the immune responses by facilitating regulatory T cell generation in the colonic mucosa [8–12]. Gut-residing microbes are also known to modify endogenous primary bile acids creating molecular species such as deoxycholate that stimulates colonic enteroendocrine cells and thus affects the regulation of the intestinal function of the host [13]. Other microbial metabolites or their host-produced derivatives, such as trimethylamine N-oxide (TMAO) and 5-aminovaleric acid betaine (5-AVAB), are known to modify specific host reactions of lipid metabolism [14–16]. Microbiota also affects the levels of intestinal and absorbed nutrients, particularly amino acids [4, 17, 18]. Microbial metabolites of amino acids, such as the indole derivatives of tryptophan, are signaling molecules with both local and systemic effects [19].

The metabolic coexistence between the animal and the bacteria begins already before birth. While it is still unclear whether small numbers of live microbes exist in the healthy fetus, hundreds of microbial metabolites originating from the dam pass through the placenta [20, 21]. Very little is known of their properties and physiological effects across developing organs of the fetus [22]. Microbe-derived aryl hydrocarbon receptor (AhR) ligands and microbially regulated retinoids are essential for fetal development of the immune system [20, 23, 24]. Maternal SCFAs are also readily transmitted to the fetus, programming the fetal metabolic and neural systems [25]. Other maternally derived microbial metabolites have been primarily studied in the context of toxicology [22]. These observations suggest that whole bacteria are not necessarily required to inflict inflammatory immune responses by the host cells [26].

**Fig. 1:**
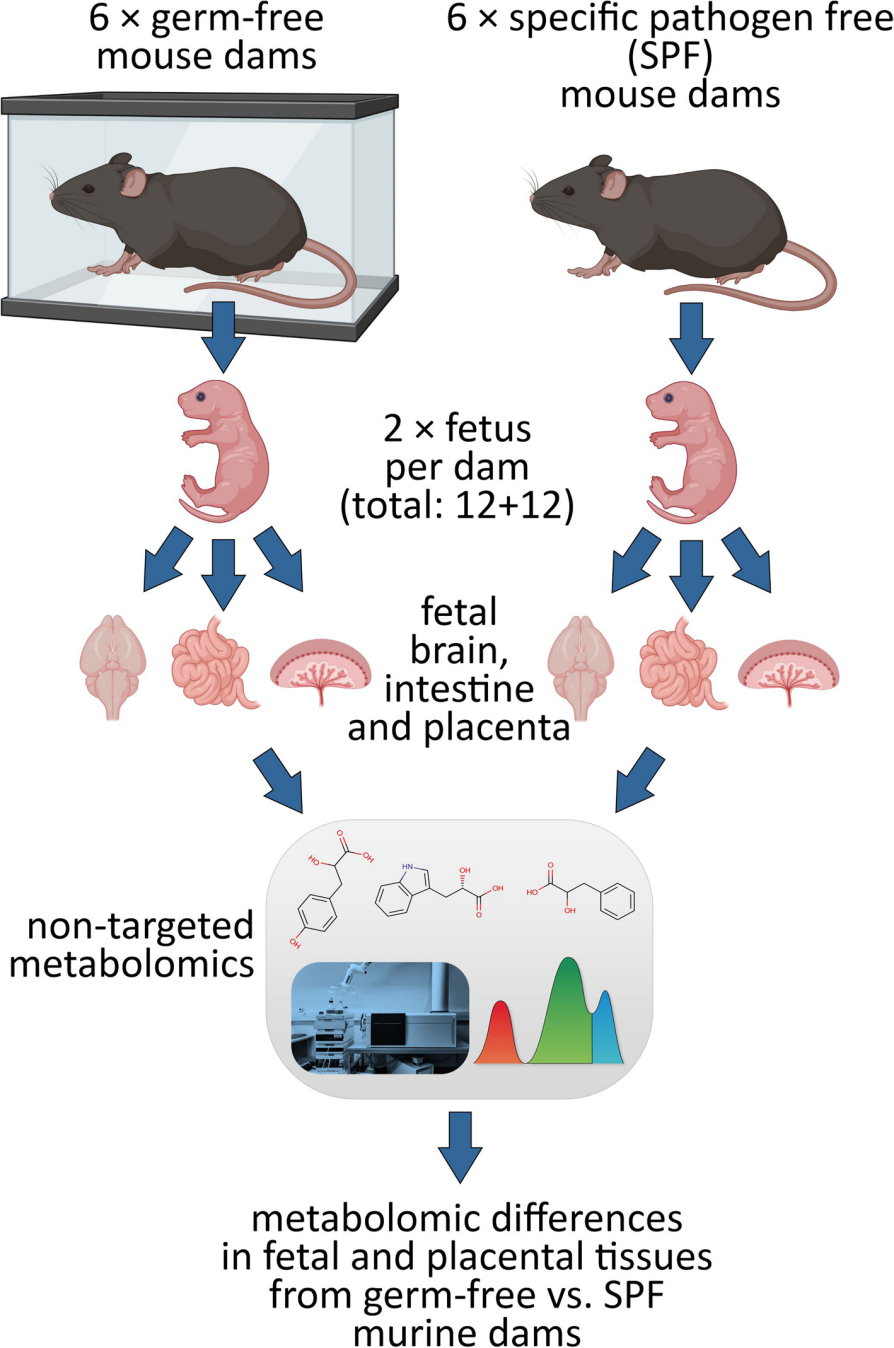
Experimental design. Fetal and placental tissues were collected from germ-free (GF) and specific pathogen free (SPF) mouse dams. The tissues were compared using non-targeted metabolomics, revealing metabolomic differences induced by maternal microbiological status in the fetuses. Figure created with Biorender.com

To examine the extent of the cross-placental transfer of microbial metabolites during pregnancy, we analyzed placenta, fetal intestine and brain samples from germ-free (GF) and specific pathogen free (SPF) murine dams using a broad non-targeted metabolomics approach (Fig. 1). Ultra-high performance liquid chromatography (UHPLC) coupled with quadrupole time-of-flight (QTOF) mass spectrometry allowed the detection of thousands of differentially abundant molecular features in the organ samples.

## Results

### Differences in all observed molecular features

**Fig. 2:**
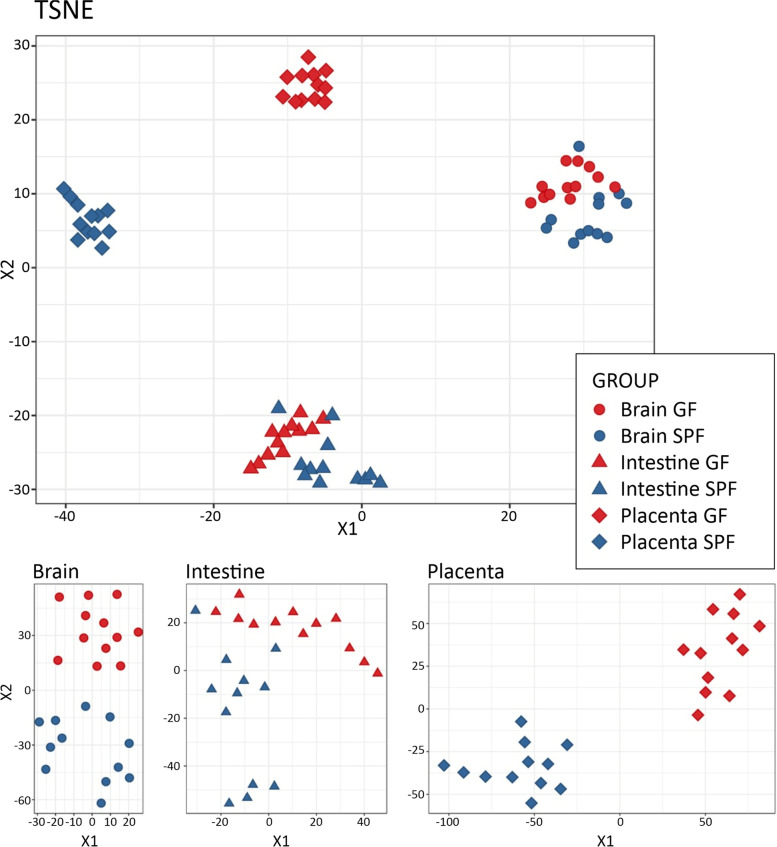
Metabolic profiles of germ-free (GF, red) and specific pathogen free (SPF, blue) placentae and fetal organs, analyzed by t-Distributed stochastic neighbor embedding (TSNE). The results are shown for the whole dataset including all the signals after data cleanup (*n* = 12,166) and separately for each organ

The non-targeted metabolomics data consisted of a total of 12,166 molecular features from four analytical modes after data cleanup. The metabolic profiles were clearly different in all studied organs (Fig. 2 and Table S1). The GF and SPF mice clustered separately in t-distributed stochastic neighbor embedding (TSNE) analysis, especially when each organ was analyzed individually (Fig. 2). The clearest separation between GF and SPF samples was observed in the placenta. The gender of the fetus did not influence the separation (not shown).

**Fig. 3:**
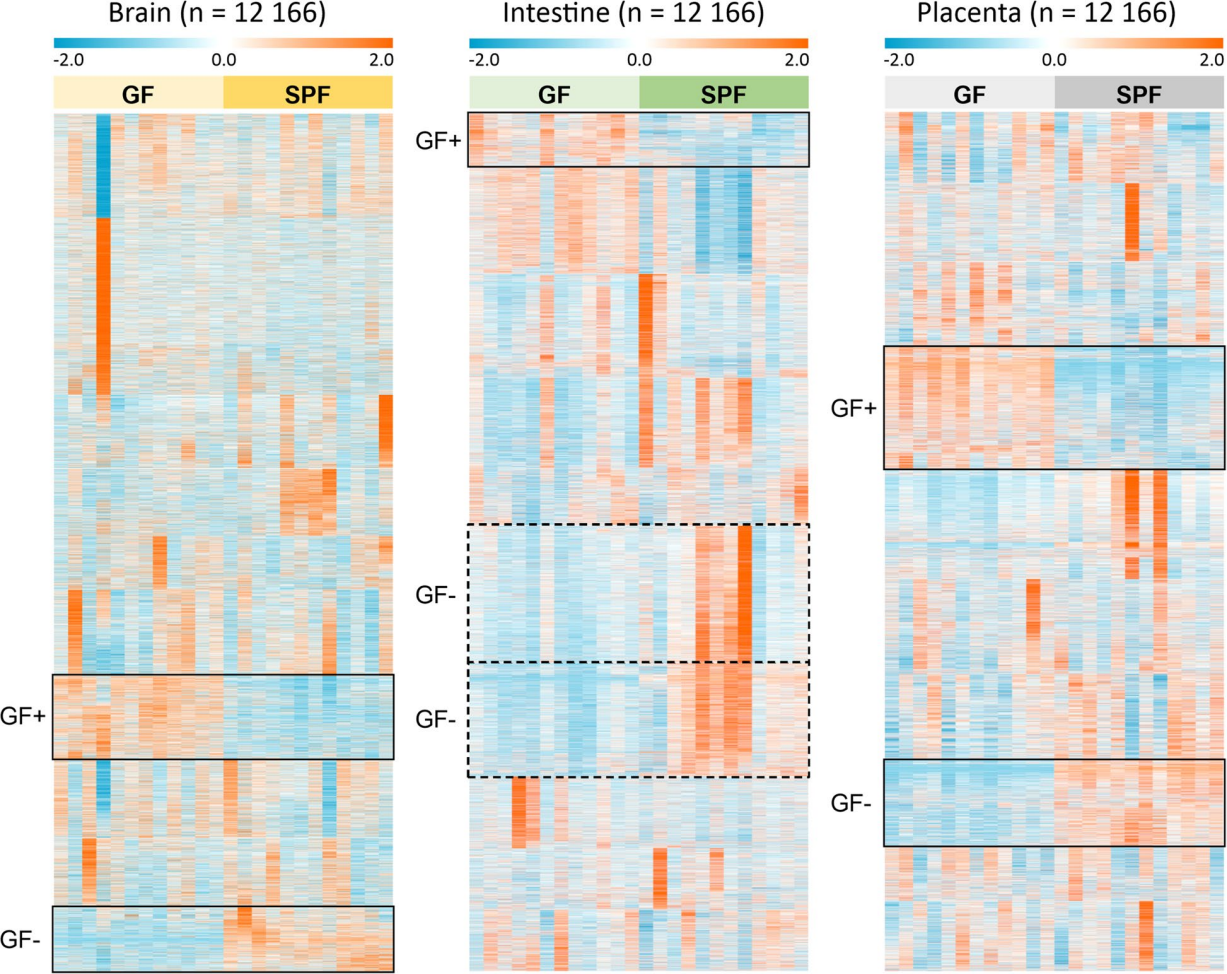
Heat map of the normalized abundances of all the signals from the four studied modes (RP+, RP−, HILIC+, HILIC−) after data cleanup (*n* = 12,166) in the brain, intestinal and placental samples. k-Means clustering (k = 10) was applied to each sample type separately to arrange the metabolites based on their similarity of the abundance between the samples. Clusters with tendency for increased or decreased abundance in the GF (germ-free) mice compared to SPF (specific pathogen free) mice are highlighted (continuous line: consistent trend across all samples, dashed line: trend with high interindividual variability). Individual signals are not aligned throughout all organ samples due to separately performed clustering

The clustering by organ is also evident in the heatmap of all observed molecular features (Fig. 3). At this level, the difference in signal abundance related to germ-free status can be observed from a few relatively small clusters of molecular features in each analyzed organ.

**Fig. 4:**
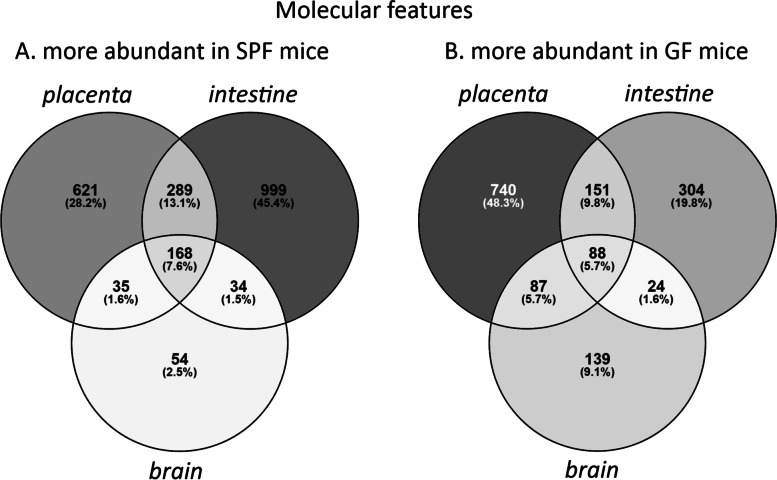
Venn diagrams showing the distribution of molecular features (*n* = 3680) which were more abundant (FDR-adjusted *p* < 0.05; Cohen’s d < − 0.8 or > 0.8) **A** in specific pathogen free mice in at least one organ, **B** in germ-free mice in at least one organ. SPF = specific pathogen free, GF = germ-free

The concentrations of 3680 molecular features differed between germ-free (GF) and specific pathogen free (SPF) mice in at least one of the organs investigated (FDR-adjusted *p* < 0.05 and Cohen’s *d* > 0.8). There were 2200 features which were more abundant in SPF mice in at least one organ (Fig. 4a). These were most numerous in the fetal intestine. One hundred sixty-eight features were more abundant in SPF mice in all three organs investigated. Similarly, 1533 features were more abundant in GF mice in at least one organ, most commonly in placenta (Fig. 4b). Eighty-eight features were more abundant in GF mice in all organs.

**Fig. 5:**
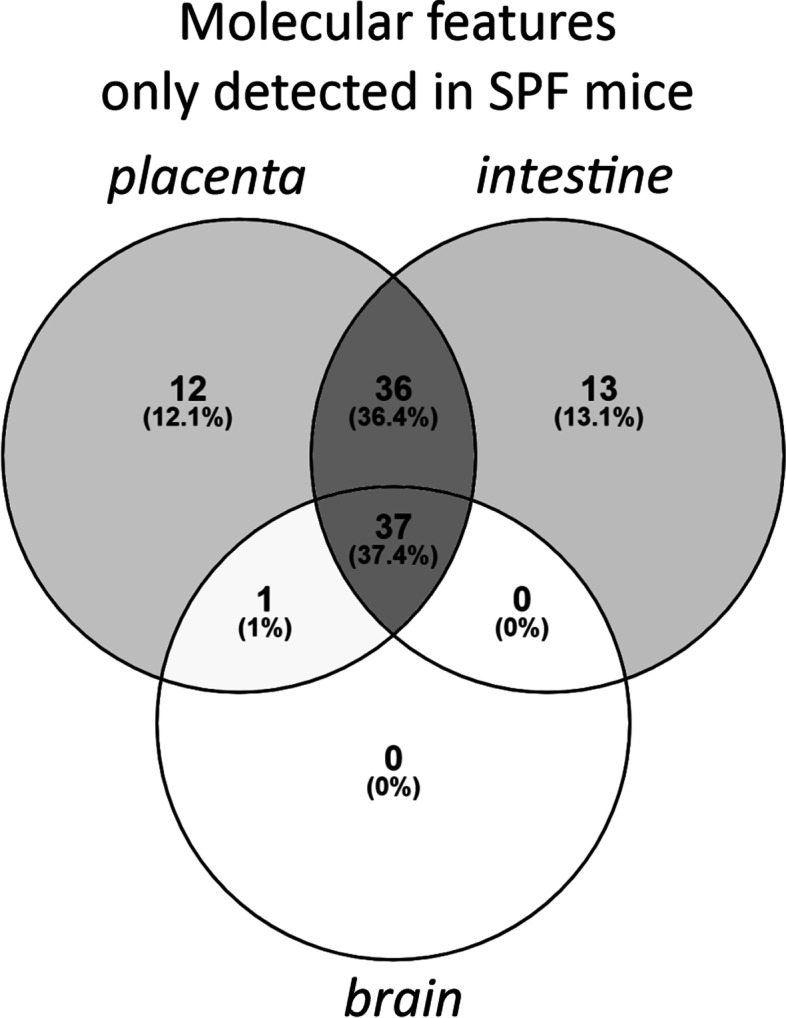
Venn diagram showing the distribution of molecular features (*n* = 99) which were observed only in specific pathogen free mice (signal-noise ratio > 5 in SPF mice and < 5 in GF mice; FDR-adjusted *p* < 0.05, Cohen’s d < − 0.8 for GF vs. SPF). SPF = specific pathogen free, GF = germ-free

A total of 99 features were only observed in SPF mice (Fig. 5). These were most commonly detected in all three SPF organs (*n* = 37), or in both placenta and fetal intestine (*n* = 36). None were detected only in both fetal organs, or only in the fetal brain. In contrast, only 6 features were observed exclusively in GF mice (not shown).

### Annotated metabolites

**Fig. 6:**
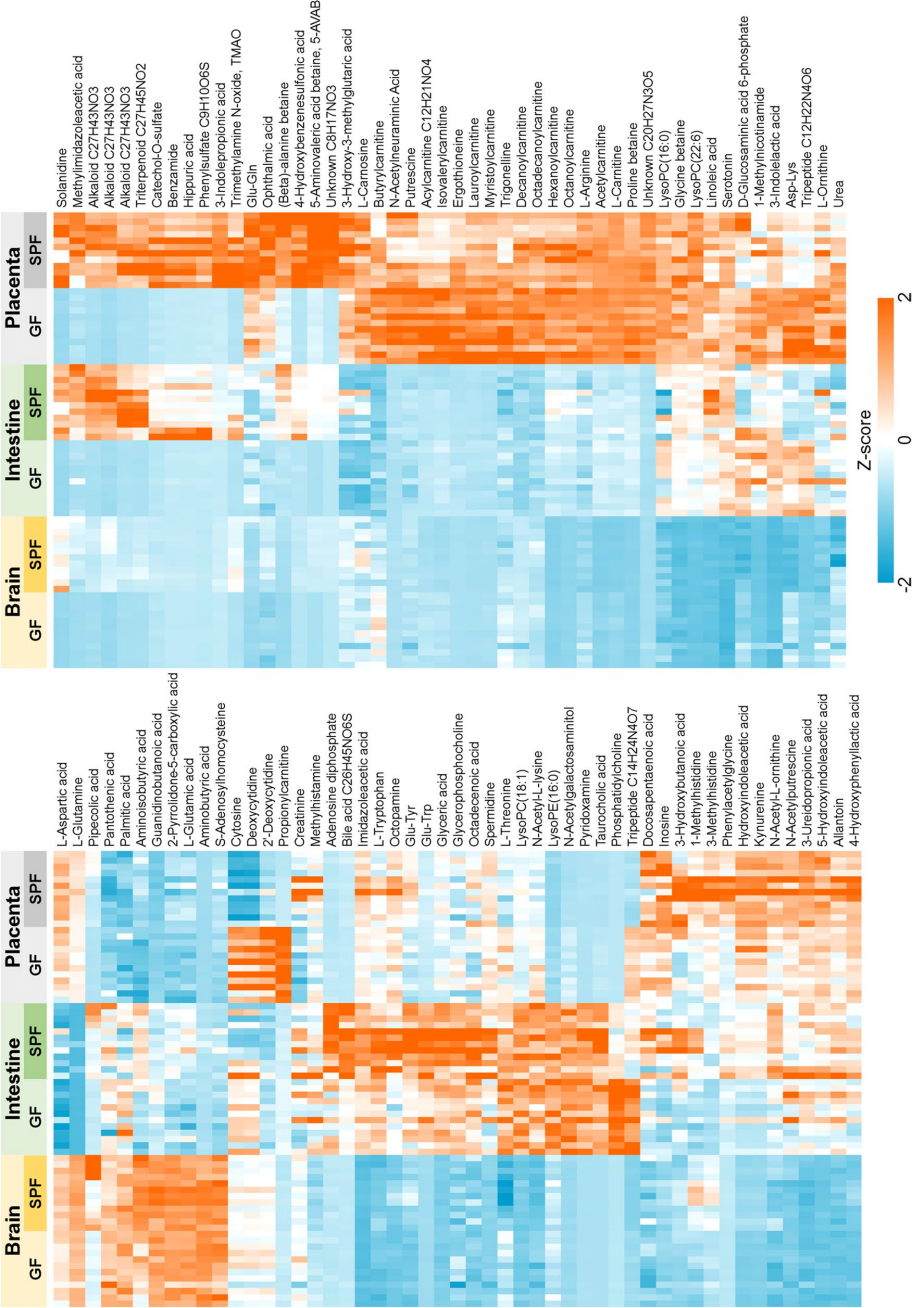
Heat map of the normalized signal abundances of the significantly differential annotated metabolites (*n* = 101) in each studied sample. A hierarchical clustering was applied to arrange the metabolites based on their similarity of the abundance between the samples

Among the differentially abundant molecular features, 101 metabolites were annotated. Out of these, 54 were identified (MSI level 1), 35 putatively annotated (level 2), 10 putatively characterized for compound class (level 3), and 2 unknowns (level 4) given a molecular formula (Table S1). A heatmap of significantly differential annotated metabolites is shown in Fig. 6. Sixty-one of these metabolites were more abundant in SPF mice in at least one organ, most of these in all three organs or in intestine and/or placenta (FDR-adjusted *p* < 0.05, Cohen’s *d* > 0.8; Figs. 6 and 8, Table S1).

**Fig. 7:**
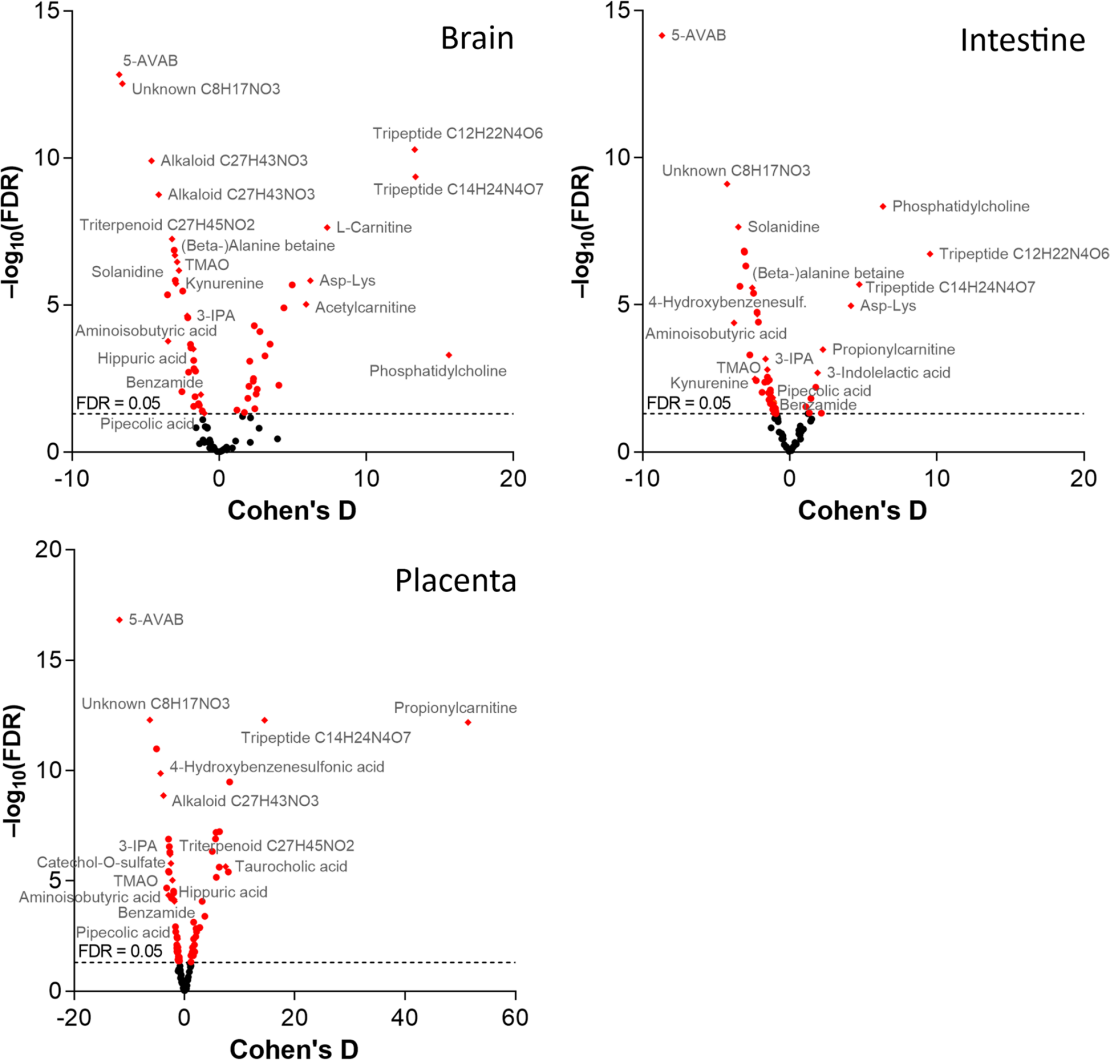
Volcano plots of the FDR-adjusted *p* values and Cohen’s d values for the significantly differential annotated metabolites (*n* = 101) in germ-free vs. specific pathogen free mice. Named compounds are represented as diamonds

**Fig. 8:**
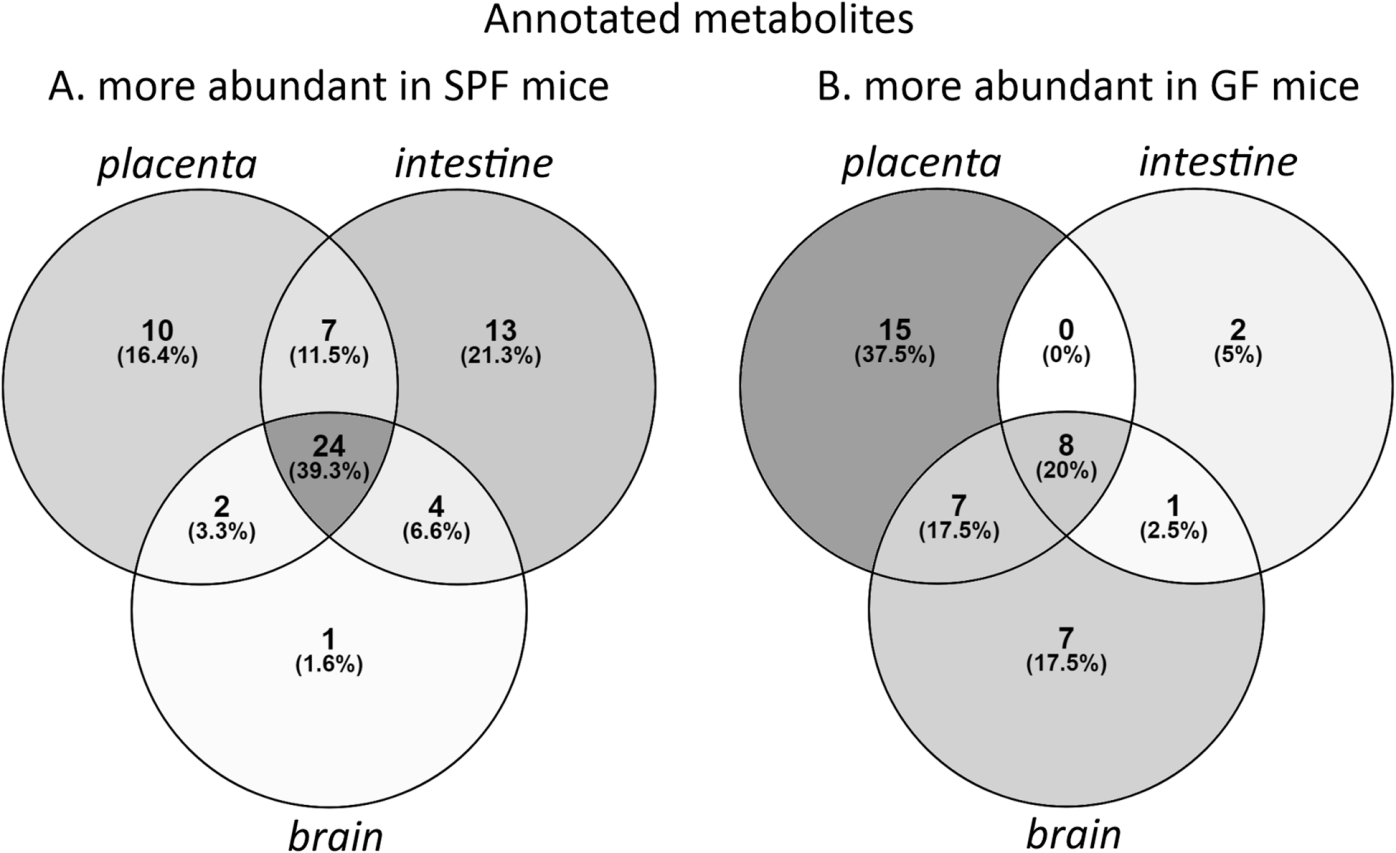
Venn diagrams showing the distribution of annotated metabolites which were more abundant (FDR-adjusted *p* < 0.05; Cohen’s d > 0.8) **A** in specific pathogen free mice in at least one organ (*n* = 61), **B** in germ-free mice in at least one organ (*n* = 40). SPF = specific pathogen free, GF = germ-free

Volcano plots of FDR-adjusted *p* values and Cohen’s *d* values for each organ are shown in Fig. 7. Twenty-five metabolites were more abundant in all three SPF organs. Of these, 5-AVAB [also known as δ-valerobetaine (δVB), N,N,N-trimethyl-5-aminovalerate (TMAV) and N,N,N-trimethyl-5-aminovaleric acid (TMAVA)] was the most affected metabolite in all three organs. Other metabolites significantly affected included TMAO, alanine / β-alanine betaine, solanidine, catechol-O-sulphate, hippuric and pipecolic acid, amino acids and their derivatives (such as kynurenine, 3-indolepropionic acid and aminoisobutyric acid) and some small peptides. Five of the annotated compounds were observed exclusively in SPF mice: benzamide, 4-hydroxybenzenesulfonic acid, two unidentified alkaloids and a triterpenoid. Forty annotated metabolites were more abundant in GF mice in at least one organ, primarily in placenta and/or brain (Fig. 8). These included several acylcarnitines, phosphatidylcholine, amino acids (such as L-threonine, L-arginine, ergothioneine and L-ornithine) and several small peptides.

### Pathway analysis

In order to predict metabolic pathways affected by the lack of microbiota in germ-free mice, we performed the MS Peaks to Pathways analysis in the MetaboAnalyst pipeline [27]. This module utilizes the mummichog algorithm and gene set enrichment analysis (GSEA), which fit the mass spectrometry peak data into known metabolic pathways without pre-existing compound annotations [27].

In terms of murine KEGG (Kyoto Encyclopedia of Genes and Genomes) and BioCyc Genome Database Collection pathways, the metabolism of several essential amino acids, as well as tRNA charging / aminoacyl-tRNA biosynthesis were significantly different in both fetal organs (Supplementary Table 2) [28, 29]. In the fetal intestine, phosphonate & phosphinate metabolism, glycolysis, caffeine metabolism, degradation of putrescine and nicotine, methionine salvage, and lipoate biosynthesis and incorporation were affected. In placenta, the affected pathways included lactose degradation, methionine salvage, nicotine degradation and folate biosynthesis.

## Discussion

This is the first study probing the effects of a complete maternal microbiota on the metabolite composition in mammalian placental and fetal tissues. We compared fetuses of germ-free (GF) and specific pathogen free (SPF) murine dams utilizing non-targeted metabolomics. The lack of maternal microbiota affected the metabolite profile of the placenta, fetal intestine and brain. A total of 2200 detected molecular features were more abundant in SPF mice in at least one organ, while more than 1500 showed higher levels in the GF mice. Approximately 100 molecular features could be detected only in the SPF organ samples. The numbers of compounds depleted in GF mice were largest in the fetal intestine and/or placenta. These observations indicate that the maternal microbiota strongly affects the host metabolism in placenta and in the fetus not only by directly producing metabolites but also pervasively impacting host physiology.

One hundred one of the differentially abundant metabolites could be annotated based on current databases. The limited number of identified metabolites, which is typical for non-targeted metabolomics study, is explained by several factors. Our metabolite identification was focused to the molecular features that showed significant difference between the experimental groups, i.e., to those metabolites that are associated with mouse gut microbiota. There are very limited reference data available for mouse gut microbiota associated metabolites when compared to, e.g., with metabolites found in human plasma or serum, reducing the number of possible identifications. Furthermore, we report each metabolite only once as an identified metabolite in the final results although a single metabolite can produce several molecular features in the data (the metabolite is measured in several modes of analysis and produces adducts and/or fragments).

Several betaines, amino acids, and their derivatives, such as pipecolic acid and small peptides, certain alkaloids, catechol-O-sulphate and hippuric acid were more abundant in SPF mice. Several acylcarnitines, some amino acids, several small peptides and phosphatidylcholine were more abundant in GF mice.

### Trimethylated compounds 5-AVAB, (β-)alanine betaine and TMAO

Considerably lower levels of the trimethylated compounds, 5-AVAB, (*β*-)alanine betaine and TMAO, were detected in all the studied organs of the GF mice. These compounds are zwitterions containing a positively charged trimethylammonium group and a negatively charged oxygen. They can act as osmoprotective substances and methyl donors in various cellular metabolic processes [30–32]. 5-AVAB is a bacterial metabolite [30, 33] with reported effects on fatty acid metabolism. It inhibits oxygen consumption due to β-oxidation of fatty acids in cultured mouse cardiomyocytes [15]. Thus, it may protect the heart tissue in ischemic conditions. The cord plasma of pre-eclamptic infants has been shown to contain increased levels of 5-AVAB [34, 35]. 5-AVAB has also been detected in the fetal mouse brain where its quantity has been shown to be strongly dependent on the presence of maternal gut microbiota [36]. Lower levels of (*β*-) alanine betaine have been detected in the intestine of GF mice than in conventional mice suggesting that it can also be produced by gut microbiota [30]. Its role in mammalian physiology is poorly known.

TMAO is the mammalian end product of dietary phosphatidylcholine, choline, and carnitine, which are first metabolized by gut microbes into trimethylamine (TMA) and further into its *N*-oxide form in the liver [37, 38]. Whilst red meat is the main dietary source for the TMAO precursors choline and carnitine, TMAO as such is abundant in seafood [39]. TMAO inhibits reverse cholesterol transport by affecting bile acid synthesis on multiple levels and increases deposition of cholesterol to arterial walls [14]. Elevated levels are associated with increased risk of cardiovascular diseases, such as atherosclerosis and thrombosis [40, 41], but a causative role of TMAO has not been definitely established [32]. A recent study indicates that TMAO and possibly also 5-AVAB have a role in development of the fetal brain [36]. More than twofold reductions of TMAO and 5-AVAB were detected in the maternal blood and fetal brain of both GF and antibiotic-treated pregnant mice relative to SPF controls. Colonization of GF dams with ingenious spore-forming bacteria increased the levels of these compounds in the fetal brain. The maternal microbiota was shown to promote fetal thalamocortical axonogenesis and the effect could be reproduced by supplementing microbiota-depleted dams with TMAO [36].

### Amino acids and related metabolites

Concentrations of several amino acids and their derivatives were significantly different in the organs of the test groups. The pathway analysis based on unannotated MS peak data further substantiated the broad impacts of maternal microbiota on amino acid metabolism and tRNA charging especially regarding essential amino acids. Earlier studies have shown that the GF status is associated with differences in the levels of several amino acids in plasma and intestine [4, 17, 18]. Notably, microbial metabolism can diversify the fates of the amino acid tryptophan. On the one hand, microbes can produce an array of microbial indole metabolites, such as indole-3-propionic acid (IPA), in the gut lumen. On the other, they can also act indirectly by modifying the host metabolism of tryptophan to its neuromodulatory metabolite serotonin by enterochromaffin cells and to kynurenine by immune and epithelial cells [13, 42–44]. Adult GF mice have been reported to exhibit lower levels of serotonin in plasma and brain but higher levels of tryptophan in plasma than in conventional mice [4, 42].

We observed lower levels of tryptophan and its metabolites serotonin, hydroxyindoleacetic acid (HIAA), kynurenine and 3-indolepropionic acid (3-IPP) in intestine or brain or both organs of the GF fetuses. The levels of serotonin and 3-IPP were also reduced in placenta. Kynurenine and HIAA can act as ligands for AhR receptors expressed on gut epithelia and many types of immune cells and may have a significant role in modifying the host mucosal immune system to promote the survival of commensal microbiota and provide protection against pathogens [44]. Modulation of the levels of tryptophan, serotonin and kynurenine by microbiota may have effects on both the enteral and central nervous systems and the immune system. Disturbances in the intestinal microbiota can affect the levels of these metabolites, which may contribute to the pathogenesis of a multitude of disorders, such as inflammatory bowel diseases, metabolic syndrome and obesity, and various neuropsychiatric disorders [19]. 3-indolepropionic acid is a strong antioxidant [45, 46]. It has been shown to regulate the intestinal barrier function by acting as a ligand to the pregnane X receptor (PXR) [47]. It also has neuroprotective functions [45] and was recently shown to promote thalamocortical axonogenesis in fetal mouse brain [36]. The changes in the levels of tryptophan metabolites in fetal intestine and brain reported here suggest that the microbial influences may begin already during fetal development.

Methylimidazoleacetic acid is the main metabolite of histamine. In this study, it was not detected in brain of the GF fetuses and its levels were also considerably lower in the other organs compared to SPF mice. Methylimidazoleacetic acid may be associated with miscarriage, potentially by the dysregulation of cytokine networks caused by imbalance in gut bacteria [48]. Another main metabolic pathway of histamine leads to the formation of imidazoleacetic acid. Its levels were decreased in the placenta and fetal intestine of the GF mice. The 4 to 6-fold lower levels in GF mice fetal organs of pipecolic acid, a degradation product of L-lysine produced by intestinal bacteria, may reflect lack of the contribution of gut microbiota to the pipecolic acid pathway of lysine catabolism in GF mice [49]. Other amino acid-related metabolites, including phenylacetylglycine, 1-methylhistidine, 3-methylhistidine, 3-hydroxybutanoic acid, Glu-Gln and Glu-Tyr, also had lower levels in the GF organs, indicating that the presence of microbiota may increase their production. Phenylacetylglycine has been identified as a gut microbial metabolite of phenylalanine with associations to health and disease in humans [50]. It has also been detected in forebrain of mice from neonatal period into adulthood [51]. Certain amino acids and small peptides, such as L-threonine and two tripeptides with unknown structure, had higher levels in the GF organs compared to SPF, suggesting that they were accumulated in the organs due to lack of microbial metabolism.

### Plant-derived metabolites catechol-O-sulfate, hippuric acid and solanidine

The mice obtain plant-derived metabolites, such as polyphenols and polyalkaloids, by ingestion of plant-based feed. These molecules can be modified in the microbial metabolism and subsequently absorbed by the host. Catechol-*O*-sulfate and hippuric acid are metabolites of dietary polyphenols, such as flavonoids and phenolic acids, via degradation by gut microbiota and subsequent sulfation or glycine conjugation in the liver [52, 53]. Hippuric acid is also produced from breakdown products of aromatic amino acids in liver, intestine and kidney for excretion into urine [4]; gut microbiota is involved in this process [54]. Levels of hippuric acid were significantly reduced in the placenta, fetal intestine and brain. Decreased levels of hippuric acid have been reported in serum of GF mice and in urine of antibiotic-treated rats [4, 54] and recently also in the brain of fetuses of GF or antibiotic-treated mice [36]. Hippuric acid is one of the metabolites reported to be present in neonatal mouse brain with amounts decreasing by age [51]. Apart from participating in removal of benzoate, a metabolite potentially toxic to mitochondria [55], hippuric acid has been suggested a role in regulation of blood glucose levels, insulin secretion by β-cells and glucose utilization in skeletal muscle [52, 56]. Solanidine is a steroidal glycoalkaloid, which is obtained via dehydroxylation from other glycoalkaloids, such as *α*-chaconine and *α*-solanine, which are abundant in potato, a component of the RM3-A-P breeding diet. Solanidine has been detected as the main metabolite in rats after oral ingestion of *α*-chaconine, and the current findings support the hypothesis that solanidine is a gut microbial metabolite of dietary glycoalkaloids in mice [57].

### Energy metabolism

Intestinal microbiota is known to affect the energy metabolism of the host. For example, SCFAs produced from dietary fibers by gut microbiota can directly contribute the host lipogenesis and gluconeogenesis. On the other hand, SCFAs and microbiota-produced secondary bile acids also act as regulators increasing insulin sensitivity and satiety thus decreasing energy consumption [58]. SCFAs may also have roles in the programming of fetal metabolism during pregnancy [59].

There were few lipids among the differentially abundant metabolites in the placenta, fetal intestine, and brain even though lipids were widely represented in the LC-MS data. Moderately higher abundances of some long-chain polyunsaturated fatty acids were measured in SPF mice and higher abundances of palmitic acid in GF mice. It is possible that more consistent and extensive effects of the microbiota on the lipid metabolite profiles would be detected in fetal tissues central in lipid metabolism, such as the liver, muscle, and adipose tissue.

We found increased levels of carnitine and various acylcarnitines in placenta and fetal organs of GF mice. These are precursors of TMAO [32], which suggests that they were not metabolized in the gut of the dam due to the lack of gut microbiota. Increased levels of carnitine could also be due to decreased levels of 5-AVAB in the GF mice, since 5-AVAB inhibits the cell membrane carnitine transporter responsible for cellular intake of carnitine [15]. Carnitine and acyl carnitines are involved in beta oxidation of fatty acids. They also have a multifaceted role in the developing brain [60].

### Unannotated molecular features undetected in GF mice

In total 3680 molecular features were differentially expressed in SPF and GF organs and 99 were not detected in GF mice. Most of these features could not yet be annotated. Unidentified metabolites strongly affected by the presence of maternal microbiota may contain hitherto unknown molecular species with potential bioactivity in the mammalian host, as was shown recently by identification of novel amino acid conjugations of bile acids by extensive bioinformatic analysis of metabolomics data from GF and SPF mice [5].

### Limitations of the study

The LC-MS method used to detect the metabolites in this study does not allow the detection of short-chain fatty acids (formic to valeric acid), many volatile organic compounds (VOCs) and molecules above 1500 Da (including lipopolysaccharides, large peptides, proteins, and nucleic acids). The SCFAs have been extensively studied already previously [6, 10–12, 43]. SCFAs are known to cross the placenta to fetal tissues and have an impact on the fetal development [61, 62].

The current databases available for metabolite identification contain the most well-known mammalian endogenous metabolites. Reference data on exogenous (e.g., plant-derived) and microbially produced metabolites is limited. Therefore, a significant proportion of molecular features in the dataset could not be annotated. A tentative characterization of the chemical class was acquired for 10 of the unknowns with the prediction of molecular formula and comparison to in silico generated MS/MS fragmentation patterns.

## Conclusions

The germ-free status of the dam strongly affects the metabolic landscape of the placenta and the developing fetus. Several known metabolites originating in microbial metabolism or combined metabolism of microbiota and the host were among the differential compounds, further highlighting the impact of gut microbiota on the host metabolism. Intriguingly, the most significantly differing metabolites included 5-AVAB and TMAO with reported effects on neural development in mice. Among these were also the tryptophan metabolite serotonin, an important regulator of neural and enteroendocrine functions, and kynurenine and HIAA that can act as effectors of the immune system through ArH receptors. Additionally, hundreds of unannotated molecular features were significantly more abundant in SPF mice or missing from GF mice, indicating that a majority of microbially processed metabolites in the fetus and placenta are still unknown. These represent a pool of compounds with potentially important bioactivities in the host and effects on the fetal development of the brain and other organ systems.

## Methods

### Fetal and placental mouse organ samples

Fetal and placental mouse organ samples from six pregnant germ-free (GF) and six specific pathogen free (SPF) C57BL/6 J dams were obtained from the EMMA Axenic Service at Instituto Gulbenkian de Ciência, Portugal. The GF dams were 3–4 months old and the SPF dams 4–5 months old. Obtaining exactly matching age groups was not possible without wasting pregnant animals, which would violate the 3R principles of animal experimentation. Significant physiological differences between groups are not likely, as both groups represent adult dams at optimal reproductive age, litter sizes were similar (in average, 8.5 pups per SPF dam and 8.33 pups per GF dam), and the sizes of the fetuses were not significantly different when controlled for litter size. The GF and SPF statuses were regularly monitored by culture and 16S qPCR (for details, see Supplementary Information: Mouse health surveillance program; the SPF dams came from the Production Area and were housed in E0 until sampling). All dams were fed identical RM3-A-P (Rat and Mouse No.3 Breeding Autoclavable) breeding diets (SDS Special Diet Services, Essex, UK), autoclaved at 121 °C. The SPF feed was autoclaved for 20 min and the GF feed for 30 min due to logistical reasons.

The dams were euthanized 18.5 days post coitum by cervical dislocation. Whole brain, intestine and placenta were collected from two fetuses per dam, to a total of 12 fetuses per experimental group. The fetal organ samples were immediately frozen in liquid nitrogen, stored at − 80 °C and transferred on dry ice to the research laboratory. In both groups, there were 7 male fetuses and 5 female fetuses.

The number of experimental animals was estimated to be sufficient based on our previous metabolomics research. No animals were excluded from the analyses. The dams were selected from the colony by the presence of copulatory plugs and pregnancy. Confounders were not specifically controlled; no obvious confounders to this study were observed.

### Sample processing

Metabolomics analyses were performed at Afekta Technologies Ltd. Frozen organ samples were thawed at 8 °C for 2 h and then weighed (approx. 100 mg) in homogenizer tubes. For the metabolite extraction, cold methanol (80% v/v) was added in a ratio of 500 μl per 100 mg of sample. The samples were homogenized (TissueLyser II bead mill, Qiagen, Hilden, Germany) using metal beads at 6 m/s for 30 s. The samples were then shaken for 5 min in room temperature and centrifuged at 14,000 rpm at 4 °C for 10 min. After the centrifugation, the samples were kept on ice for 5 to 10 min, after which the supernatant was filtered (Acrodisc 0.2 μm PTFE membrane, Pall, Port Washington, NY, USA) into HPLC vials for analysis. The pooled quality control (QC) sample was prepared by collecting 20 μl from each sample vial and combining the material to two vials.

The laboratory staff was blinded to the identity of the samples and was not aware of the experimental setup or grouping. The samples were randomized to analysis batches.

### LC–MS analysis

The samples were analyzed by liquid chromatography–mass spectrometry (LC-MS), consisting of a 1290 Infinity Binary UPLC coupled with a 6540 UHD Accurate-Mass Q-TOF (Agilent Technologies Inc., Santa Clara, CA, USA), as described previously [63]. In brief, a Zorbax Eclipse XDB-C18 column (2.1 × 100 mm, 1.8 μm; Agilent Technologies) was used for the reversed-phase (RP) separation and an Acquity UPLC BEH amide column (Waters Corporation, Milford, MA, USA) for the HILIC separation. After each chromatographic run, the ionization was carried out using jet stream electrospray ionization (ESI) in the positive and negative mode, yielding four data files per sample. The collision energies for the MS/MS (tandem mass spectrometry) analysis were selected as 10, 20 and 40 V, for compatibility with spectral databases.

### Data analysis

Peak detection and alignment were performed in MS-DIAL ver. 4.00 [64]. For the peak collection, *m/z* values between 50 and 1500 and all retention times were considered. The amplitude of minimum peak height was set at 2000. The peaks were detected using the linear weighted moving average algorithm. For the alignment of the peaks across samples, the retention time tolerance was 0.05 min, and the *m/z* tolerance was 0.015 Da. The heatmaps were produced with Multiple Experiment Viewer (MeV) version 4.9.0. MetaboAnalyst 4.0 was used for the pathway analysis of the annotated metabolites [27]. Data clean-up (for each mode separately) and statistics (for all signals remaining after clean-up) were performed in R version 3.5.1. Molecular features were only kept if they met all the following quality metrics criteria: low number of missing values, present in more than 70% of the QC samples, present in at least 60% of samples in at least one study group, RSD* (the non-parametric version of relative standard deviation) below 20%, D-ratio* (non-parametric measure of the spread of the QC samples compared to the biological samples) below 10%. In addition, if either RSD* or D-ratio* was above the threshold, the features were still kept if their classic RSD, RSD* and basic D-ratio were all below 10%. Low-quality features were flagged and discarded from statistical analyses. Drift correction was applied to the data.

The cleaned data matrices of the four modes were combined before imputation. Features were then imputed using random forest imputation with an OOB error of 0.009. QC samples were removed prior to imputation to prevent them from biasing the procedure. Differential features between the treatment (GF) and control (SPF) were determined using a simple linear model (Student’s *t*-test) fit separately for each feature. The results were adjusted for multiple comparisons using Benjamini–Hochberg false discovery rate (FDR). FDR-adjusted *p* values (*q* values) below 0.05 were considered significant.

For the MS Peaks to Pathways analysis in MetaboAnalyst 4.0 [27], the data was first normalized by medians, cube root transformed, automatically scaled, and parametric statistical significances calculated with equal variances and adjusted *p* value (FDR) cutoff 0.05. In Peaks to Pathways, the molecular weight tolerance was set to 10 ppm, primary ions enforced, and adducts set based on the experimental data. Gene Set Enrichment analysis [65] and Mummichog version 1.0.10 [66] were used.

### Compound identification

The chromatographic and mass spectrometric characteristics (retention time, exact mass, and MS/MS spectra) of the significantly differential molecular features were compared with entries in an in-house standard library and publicly available databases, such as METLIN and HMDB, as well as with published literature. The annotation of each metabolite, and the level of identification was given based on the recommendations published by the Chemical Analysis Working Group (CAWG) Metabolomics Standards Initiative (MSI): level 1 refers to confirmed identifications based on reference standards analyzed with the same instrument with identical conditions; level 2 means putative annotations with matching *m/z* and MS/MS fragmentation spectra with publicly available databases; level 3 signifies a putative characterization of compound class based on the observed physicochemical characteristics of the molecular feature; and level 4 covers all the remaining (unknown) signals [67]. Level 1–3 annotations included comparison to reference MS/MS spectra. The small peptides were annotated based on exact mass and comparison of the MS/MS spectra with publicly available databases, such as METLIN. The order of the amino acid residues could be deduced from the MS/MS spectra. Level 2 annotation was also given if MS/MS data was not available but the retention time and calculated molecular formula matched with that of a reference standard.

## Supplementary Information

**Additional file 1.**

**Additional file 2.**

**Additional file 3.**

## Data Availability

The metabolomics dataset for this study can be found at EUDAT: 10.23728/b2share.4be0ea9f87b84a06be960d6a1c4b0b42.

## Abbreviations

3-IPP: 3-indolepropionic acid
5-AVAB, also known as δ-valerobetaine (δVB), N,N,N-trimethyl 5-aminovalerate (TMAV) and N,N,N-trimethyl-5-aminovaleric acid (TMAVA): 5-aminovaleric acid betaine
AhR: Aryl hydrocarbon receptor
CAWG: Chemical Analysis Working Group
GSEA: Gene set enrichment analysis
GF: Germ-free
HIAA: Hydroxyindoleacetic acid
IPA: Indole-3-propionic acid
ESI: Jet stream electrospray ionization
KEGG: Kyoto Encyclopedia of Genes and Genomes
LC-MS: Liquid chromatography–mass spectrometry
MS: Mass spectrometry
MSI: Metabolomics Standards Initiative
PXR: Pregnane X receptor
QTOF: Quadrupole time-of-flight
QC: Quality control
RM3-A-P: Rat and Mouse No.3 Breeding Autoclavable
SPF: Specific pathogen free
SCFAs: Short chain fatty acids
MS/MS: Tandem mass spectrometry
TSNE: t-Distributed stochastic neighbor embedding
TMA: Trimethylamine
TMAO: Trimethylamine N-oxide
UHPLC: Ultra-high performance liquid chromatography
