# Supplementary material for: Maternal microbiota-derived metabolic profile in fetal murine intestine, brain and placenta
Source: BMC Microbiol. 2022 Feb 7;22:46. doi: 10.1186/s12866-022-02457-6 (PMC8819883; doi:10.1186/s12866-022-02457-6)
Supplement: Supplementary file 3 — Additional file 3. [file 12866_2022_2457_MOESM3_ESM.pdf]

GULBENKIAN SCIENCE

Health Surveillance Program

Mouse Facility

Instituto Gulbenkian de Ciência

Testing laboratories: IDEXX BioAnalytics
Location: Production, Experimental 0, and 1
Housing: IVC and Filter cages
Sentinels: Balb/c
Species: mouse (several strains)

Agents tested at IGC (mouse)

	Production + E0			E1	
Sample	Annual SOPF	Quarterly SOPF	Sample	Annual SPF	Quarterly SPF
Blood	MPV	MPV	Blood	MPV	MPV
Blood	MVM	MVM	Blood	MVM	MVM
Blood	TMEV	TMEV	Blood	TMEV	TMEV
Blood	Pneumocystis murina	-	Blood	-	-
Blood	MCMV	-	Blood	MCMV	-
Blood	Sendai	-	Blood	Sendai	-
Blood	Clostridium piliforme	-	Blood	Clostridium piliforme	-
Blood	Mycoplasma pulmonis	-	Blood	Mycoplasma pulmonis	-
Blood	Ectromelia	-	Blood	Ectromelia	-
Blood	LCMV	-	Blood	LCMV	-
Blood	MAV1	-	Blood	MAV1	-
Blood	MAV2	-	Blood	MAV2	-
Blood	PVM	-	Blood	PVM	-
Blood	REO3	-	Blood	REO3	-
	17 agents	6 agents		16 agents	6 agents

	Production + E0			E1	
Sample	Annual SOPF	Quarterly SOPF	Sample	Annual SPF	Quarterly SPF
feces	Pasteurella pneumotropica biotype Jawetz	Pasteurella pneumotropica biotype Jawetz	feces	Pasteurella pneumotropica biotype Jawetz	Pasteurella pneumotropica biotype Jawetz
feces	Pasteurella pneumotropica biotype Heyl	Pasteurella pneumotropica biotype Heyl	feces	Pasteurella pneumotropica biotype Heyl	Pasteurella pneumotropica biotype Heyl
feces	Streptococcus pneumoniae	Streptococcus pneumoniae	feces	Streptococcus pneumoniae	Streptococcus pneumoniae
feces	Streptococcus sp. B hemolytic (Group A)	Streptococcus sp. B hemolytic (Group A)	feces	Streptococcus sp. B hemolytic (Group A)	Streptococcus sp. B hemolytic (Group A)
feces	Streptococcus sp. B hemolytic (Group B)	Streptococcus sp. B hemolytic (Group B)	feces	Streptococcus sp. B hemolytic (Group B)	Streptococcus sp. B hemolytic (Group B)
feces	Streptococcus sp. B hemolytic (Group C)	Streptococcus sp. B hemolytic (Group C)	feces	Streptococcus sp. B hemolytic (Group C)	Streptococcus sp. B hemolytic (Group C)
feces	Streptococcus sp. B hemolytic (Group G)	Streptococcus sp. B hemolytic (Group G)	feces	Streptococcus sp. B hemolytic (Group G)	Streptococcus sp. B hemolytic (Group G)
feces	Helicobacter spp.	Helicobacter spp.	feces	Helicobacter spp.	Helicobacter spp.
feces	(Helicobacter bilis)	(Helicobacter bilis)	feces	(Helicobacter bilis)	(Helicobacter bilis)
feces	(Helicobacter ganmani)	(Helicobacter ganmani)	feces	(Helicobacter ganmani)	(Helicobacter ganmani)
feces	(Helicobacter hepaticus)	(Helicobacter hepaticus)	feces	(Helicobacter hepaticus)	(Helicobacter hepaticus)
feces	(Helicobacter mastomyrinus	(Helicobacter mastomyrinus	feces	(Helicobacter mastomyrinus	(Helicobacter mastomyrinus
feces	(Helicobacter rodentium)	(Helicobacter rodentium)	feces	(Helicobacter rodentium)	(Helicobacter rodentium)
feces	(Helicobacter typhlonius)	(Helicobacter typhlonius)	feces	(Helicobacter typhlonius)	(Helicobacter typhlonius)
feces	Giardia muris	Giardia muris	feces	Giardia muris	Giardia muris
feces	Aspiculuris tetraptera	Aspiculuris tetraptera	feces	Aspiculuris tetraptera	Aspiculuris tetraptera
feces	Spironucleus muris	Spironucleus muris	feces	Spironucleus muris	Spironucleus muris
feces	Syphacia muris	Syphacia muris	feces	Syphacia muris	Syphacia muris
feces	Syphacia obvelata	Syphacia obvelata	feces	Syphacia obvelata	Syphacia obvelata
feces	Cryptosporidium spp.	Cryptosporidium spp.	feces	Cryptosporidium spp.	Cryptosporidium spp.
feces	Citrobacter rodentium	-	feces	Citrobacter rodentium	-
feces	Salmonella sp.	-	feces	Salmonella sp.	-
feces	Streptobacillus moniliformis	-	feces	Streptobacillus moniliformis	-
feces	Corynebacterium kutscheri	-	feces	Corynebacterium kutscheri	-
feces	Bordetella bronchiseptica	-	feces	-	-
feces	Proteus mirabilis	-	feces	-	-
feces	Staphylococcus aureus	Staphylococcus aureus	feces	-	-
feces	Klebsiella oxytoca	Klebsiella oxytoca	feces	-	-
feces	Klebsiella pneumoniae	Klebsiella pneumoniae	feces	-	-
feces	Pseudomonas aeruginosa	Pseudomonas aeruginosa	feces	-	-
feces	Entamoeba muris	Entamoeba muris	feces	-	-
feces	Tritrichomonas muris	Tritrichomonas muris	feces	-	-
fur swab	Myocoptes	Myocoptes	fur swab	Myocoptes	Myocoptes
fur swab	Radfordia/Myobia	Radfordia/Myobia	fur swab	Radfordia/Myobia	Radfordia/Myobia
fur swab	Corynebacterium bovis	-	fur swab	-	-
	35 agents	28 agents		26 agents	22 agents
